# Rhabdomyolysis Related to Red Yeast Rice Ingestion

**DOI:** 10.7759/cureus.33532

**Published:** 2023-01-09

**Authors:** Sara Santos, Sara Gomes, Inês Carvalho, Inês Bonito, Célia Carmo

**Affiliations:** 1 Internal Medicine, Centro Hospitalar Barreiro-Montijo, Barreiro, PRT

**Keywords:** statin, dyslipidemia, monacolin k, red yeast rice, rhabdomyolysis

## Abstract

Red yeast rice is made by fermenting white rice with the fungus *Monascus purpureus*. It has lipid-lowering effects due to the presence of monacolin, produced by the fungus, and therefore shares the same biological and side effects as a statin, namely it may cause rhabdomyolysis. In this clinical case study, we report the case of a 50-year-old woman suffering from a sudden onset of chest discomfort and generalized myalgia. Laboratory findings were consistent with the diagnosis of rhabdomyolysis. The patient had been recently diagnosed with dyslipidemia in primary care, and decided to start eating red yeast rice, without informing clinicians. Clinical improvement was rapidly noticed after hydration, and blood sample results returned to normal. Awareness should be raised for the regulation of these products, as their consumption is rising, and patients are unaware of their potential side effects.

## Introduction

Nowadays, with the growing trends related to leading a healthier lifestyle, the market for natural health products is on the rise [1-2]. Red yeast rice is known for its lipid-lowering effects, due to the presence of varying amounts of natural monacolins, mainly monacolin K, which has the same chemical structure as lovastatin [3]. Both act by inhibiting HMG-CoA (5-hydroxy-3-methylglutaryl-coenzyme A) reductase and seem to share the same adverse effects [3-4]. One of its possible adverse effects is the development of rhabdomyolysis, which usually has a benign course following hydration and removal of the insulting agent [4]. Here, we describe the case of a middle-aged woman which, due to the recent diagnosis of dyslipidemia, promptly started the intake of red yeast rice. Doing so, led to the development of rhabdomyolysis without any major complication. We consider it relevant to report this case because both clinicians and patients should be aware of the potential adverse effects of these products and possible drug interactions. Clinicians should also be aware that patients often omit these products from their usual medication, as they do not recognize it as potentially harmful.

## Case presentation

The patient is a female, 50-year-old, with a known past medical history of arterial hypertension and depression, under the following medication: propranolol 10 mg, venlafaxine 200 mg and diazepam 5mg, once a day each. The patient sought medical help, after a sudden onset of chest discomfort and generalized myalgia, denying any other accompanying symptoms. On admission the vital signs were stable, she had no fever, and no relevant physical findings. Relevant laboratory findings on admission and on follow-up are shown in Tables 1-2.

**Table 1:** Blood analysis. Rhabdomyolysis pattern with normal liver and renal function tests. Thyroid function is also normal.

Laboratory tests	Results	On follow-up	Reference values
Creatine phosphokinase (IU/L)	5163	81	30–145
Creatine phosphokinase – MB (ng/mL)	2.30	1.80	0–4.9
Lactate dehydrogenase (IU/L)	330	146	120–246
Myoglobin (ng/mL)	221	50	25–72
Troponin I (pg/mL)	2.1	1.3	<15.6
Urea (mg/dL)	34	41	10–50
Creatinine (mg/dL)	0.64	0.77	0.55–1.02
Alanine transaminase (IU/L)	27	28	<55
Aspartate transaminase (IU/L)	20	23	<34
Thyroid-stimulating hormone (mU/L)	1.74	-	0.36–4.94
Free thyroxine 4 (ng/dL)	0.91	-	0.7–1.48

**Table 2:** Urinalysis results on admission and on follow-up.

Urinalysis	Results	On follow-up	Reference values
Color	Pale yellow	Pale yellow	-
Clarity	Clear	Clear	-
pH	6.5	6.0	-
Specific gravity	1.008	1.011	-
Glucose	Negative	Negative	Negative
Blood	Negative	Negative	Negative
Ketones	Negative	Negative	Negative
Protein	Negative	Negative	Negative
Urobilinogen	Negative	Negative	Negative
Bilirubin	Negative	Negative	Negative
Leukocyte esterase	Negative	Negative	Negative
Nitrite	Negative	Negative	Negative
Urine microscopy			
White blood cells	2 to 5 per high-power field	8 to 5 per high-power field	0 to 5 per high-power field
Red blood cells	0 to 4 per high-power field	0 to 4 per high-power field	0 to 4 per high-power field
Squamous epithelial cells	None	Some	None

Due to high levels of serum creatine phosphokinase (CK), the patient was asked again about her habits, daily exercise activities, and recent intake of unprescribed health products. The patient then mentioned she had been recently diagnosed with dyslipidemia by her general practitioner and had decided not to start oral statins, opting for lifestyle modifications, engaging in regular physically activity and diet restrictions. In this process, the patient bought red rice yeast, and had been eating it at least for the three days prior to admission. 

**Figure 1:**
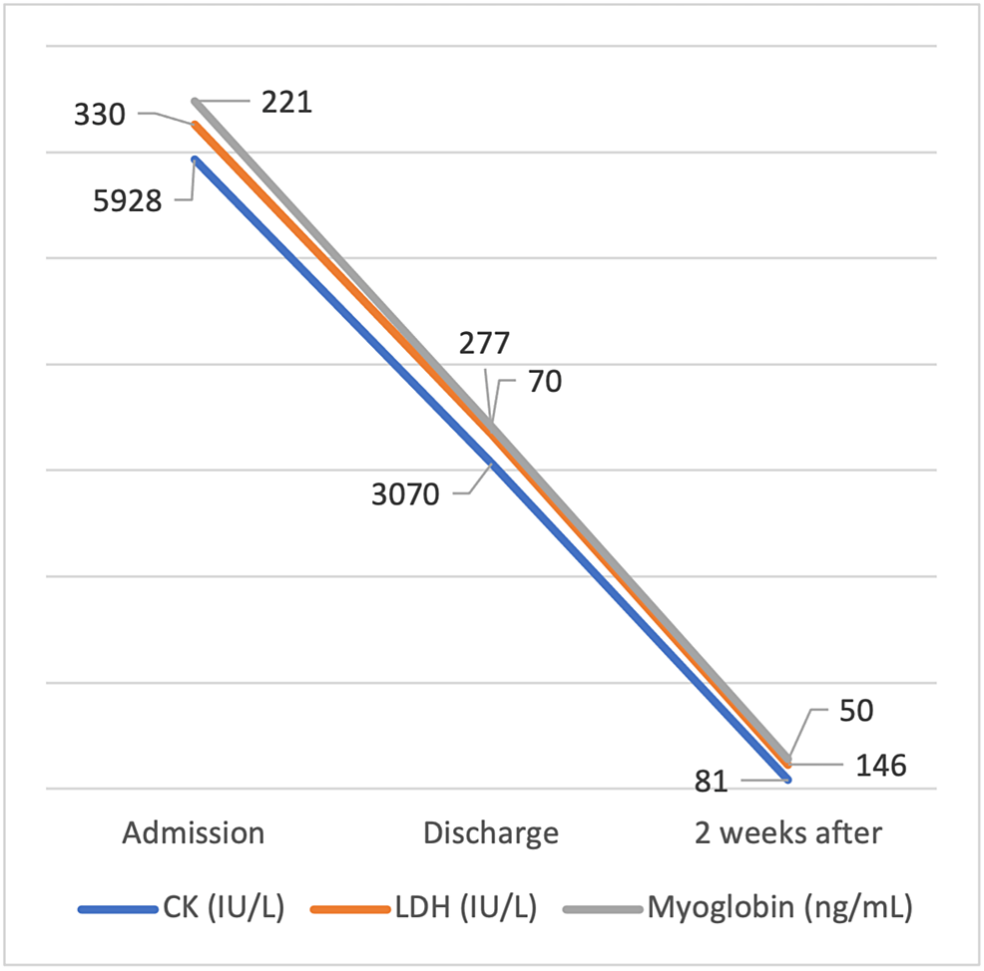
Serum CK, LDH, and myoglobin decreasing levels over a 2-week period. LDH, lactate dehydrogenase; CK, creatine phosphokinase

**Figure 2:**
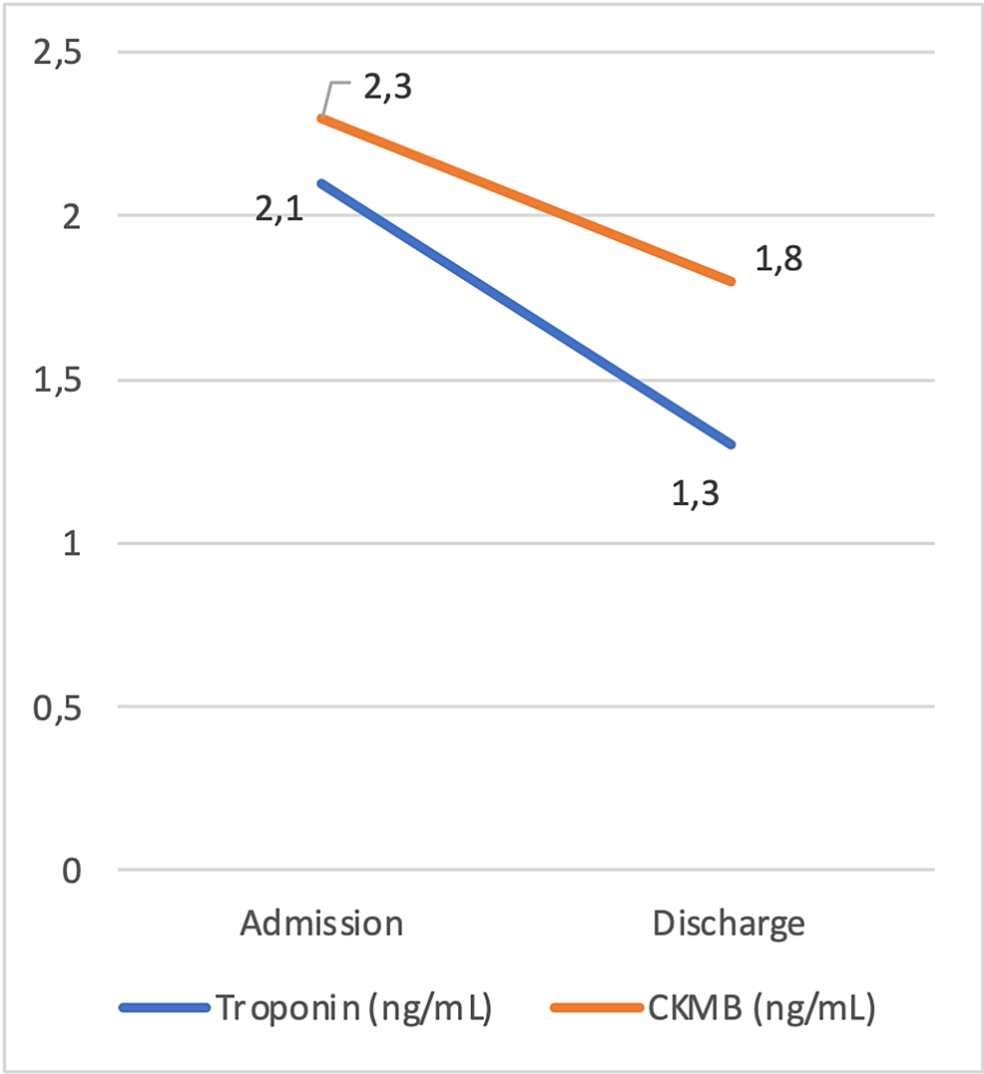
Serum troponin and CKMB levels. Evolution from admission to discharge. CKMB, creatine phosphokinase MB

The patient was placed under IV fluids and monitored for liver or kidney injury as serum CK values dropped. Apart from hydration, alkalinizing the urine is recommended in the case of higher levels of CK, to prevent myoglobin deposition and damage to the renal tubules. As the analytical evolution was favorable, the patient was discharged, advised to stop red yeast rice consumption and to keep hydrated. The patient was re-evaluated 2 weeks later, maintaining clinical and analytical stability, as shown in Figures 1-2.

 

## Discussion

According to Lin et al., the regular intake of red yeast rice 600 mg twice daily reduces serum concentrations of low-density lipoprotein cholesterol ( LDL-C) by 27.7%, total cholesterol by 21.5%, triglycerides by 15.8%, and apolipoprotein B by 26%, after 8 weeks [5]. Compared to a daily dose of low intensity statin, such as 20 mg of lovastatin, in which case the LDL-C lowering efficacy has been reported to be less than 30% in average, the results are similar [6-7].

Red yeast rice efficacy is not only due to the presence of monacolins but also because it contains ergosterol, amino acids, flavonoids, trace elements, alkaloids, sterols, isoflavones, and monounsaturated fatty acids which improve the lipid profile [8]. Its ingestion could be a relatively safe therapeutic option in dosages of 800-4800 mg/day, if a highly purified and certified red yeast rice is used [8]. Unfortunately, its safety profile is dubious, as dietary supplements are not as adequately tested or regulated as medical drugs, hence the quantity and quality of ingredients vary [3, 9]. This variation is enough to provoke undesired side effects like acute kidney injury and hepatotoxicity with a seemingly regular dose [10-11]. It also raises concern regarding drug interactions, in particular those that cause inhibition of cytochrome P450 3A4, potentiating the side effects of monacolin [4, 7]. In this case, fortunately, our patient’s medication did not significantly interact with monacolin.

We would like to highlight, besides drug interactions, two medical conditions which increase the susceptibility to statin induced rhabdomyolysis and should be considered in this case. These are untreated hypothyroidism, for which the screening was normal, and vitamin D deficiency [12-13]. Other predisposing factors that can aggravate the toxicity of myoglobin are older age, frailty, multisystem diseases, impaired liver function, and impaired kidney function (such as chronic kidney disease) [12].

In the case presented, the patient suffered complaints of myalgia due to rhabdomyolysis without any other organ disfunction and had a favorable clinical evolution following hydration and drug discontinuation. Alkalizing the urine is recommended as part of the treatment for rhabdomyolysis, if CK values are above 6000 IU/L and if in the presence of risk factors to kidney tubular lesion [14]. This patient presented borderline CK values on admission, and no other risk factors. Hence, clinicians opted for vigorous IV hydration only.

Since there is a growing tendency for overall consumption of natural products, awareness should be raised for potential side effects, drug interactions, and the need for regulation [2-3]. Clinicians, as vessels for health education [15], should ask patients specifically about the consumption of these products, as they might not recognize it as part of their regular medication or its potential side effects, thereby omitting it and consequently interfering with the diagnostic approach and unknowingly continue exposure to these substances.

## Conclusions

This case regards a patient with complaints of myalgia due to rhabdomyolysis induced by the consumption of red yeast rice, without any other organ disfunction, and with a favorable clinical outcome. Although less effective than statins, serum cholesterol levels lower with regular intake of red yeast rice. Awareness should be raised for potential side effects, drug interactions, and the need to regulate these products. Thus, clinicians should ask patients specifically about the consumption of these products, as they tend to omit it from their regular medication and help provide adequate health education to consumers.
